# Molecular evolution of a central region containing B cell epitopes in the gene encoding the p67 sporozoite antigen within a field population of *Theileria parva*

**DOI:** 10.1007/s00436-015-4358-6

**Published:** 2015-02-13

**Authors:** Isaiah Obara, Seitzer Ulrike, Tony Musoke, Paul R. Spooner, Ahmed Jabbar, David Odongo, Stephen Kemp, Joana C. Silva, Richard P. Bishop

**Affiliations:** 1 International Livestock Research Institute (ILRI), P.O. Box 30709, Nairobi, 00100 Kenya; 2 Veterinary Infection Biology and Immunology, Research Center Borstel, Parkallee 22, 23845 Borstel, Germany; 3 Institute for Genome Sciences, Department of Microbiology and Immunology, University of Maryland School of Medicine, Baltimore, 21201 MD USA

**Keywords:** B cell epitopes, *Theileria parva*, *p67* gene

## Abstract

Protective immunity induced by the infective sporozoite stage of *Theileria parva* indicates a potential role for antibodies directed against conserved serologically reactive regions of the major sporozoite surface antigen *p67* in vaccination to control the parasite. We have examined the allelic variation and determined the extent of B cell epitope polymorphism of the gene encoding *p67* among field isolates originating from cattle exposed to infected ticks in the Marula area of the rift valley in central Kenya where the African cape buffalo (*Syncerus caffer*) and cattle co-graze. In the first of two closely juxtaposed epitope sequences in the central region of the p67 protein, an in-frame deletion of a seven-amino acid segment results in a truncation that was observed in parasites derived from cattle that co-grazed with buffalo. In contrast, the variation in the second epitope was primarily due to nonsynonymous substitutions, resulting in relatively low overall amino acid conservation in this segment of the protein. We also observed polymorphism in the region of the protein adjacent to the two defined epitopes, but this was not sufficient to provide statistically significant evidence for positive selection. The data indicates that B cell epitopes previously identified within the *p67* gene are polymorphic within the Marula field isolates. Given the complete sequence identity of the *p67* gene in all previously characterized *T. parva* isolates that are transmissible between cattle by ticks, the diversity observed in *p67* from the Marula isolates in combination with the clinical reaction of the infected cattle is consistent with them originating from ticks that had acquired *T. parva* from buffalo.

## Introduction

*Theileria parva* is an apicomplexan protozoan parasite of cattle that is transmitted by the tick *Rhipicephalus appendiculatus* and is the causal agent of East Coast fever, a frequently fatal disease of cattle in eastern and southern Africa (Norval et al. 1992). The tick inoculates *T. parva* sporozoites that are infective to the mammalian host, and these differentiate rapidly into an intracellular schizont that immortalizes bovine lymphocytes; subsequently, *T. parva* differentiates into a piroplasm stage that is infective to erythrocytes. Transmission to cattle can originate from ticks that have fed on African Cape buffalo (S*yncerus caffer*), the major wildlife reservoir of *T. parva*, in which case, there is a different clinical syndrome, known as “corridor disease” involving low levels of schizont parasitosis and piroplasm parasitemia. Transmission by ticks that have previously fed on other cattle results in typical East Coast fever symptoms with higher levels of parasites. Previous studies using both variable number tandem repeats (VNTRs) and antigen genes have revealed genetic differences between parasites transmissible among cattle and those found in buffalo (Oura et al. 2011; Pelle et al. 2011), suggesting that the two sets of parasites represent distinct populations.

*T. parva* has been the subject of more than 40 years of research aimed at development of a recombinant vaccine using antigens derived from both the sporozoite and schizont stages (reviewed in Morrison 2009; McKeever et al. 1999). The closest approach to an effective anti-sporozoite vaccine to date has used a recombinant version of p67, the major sporozoite surface antigen of *T. parva*. The p67 protein induces a consistent level of 70 % efficacy against either heterologous or homologous needle challenge using sporozoite stabilates in the laboratory (Musoke et al. 1992; Bishop et al. 2003) and reduced severe disease by approximately 45 % in field trials where cattle were exposed to infective ticks (Musoke et al. 2005). Five regions of the p67 protein sequence that are reactive with anti-sporozoite monoclonal antibodies and also correlate with the results of an in vitro parasite-neutralizing assay have been identified using Pepscan analysis (Nene et al. 1999). The central region of the *p67* gene includes two closely linked epitopes that are the target of host B cell responses and whose sequences in the *T. parva* Muguga reference stock are ^169^ TKEEVPPADLSDQVP ^183^ and ^209^ LQPGKTS ^215^. These are subsequently referred to as epitopes 1 and 2, respectively.

While the *p67* gene is variable among buffalo-derived isolates, the predicted p67 protein appears to be invariant in cattle-derived stocks of *T. parva* that are transmissible between cattle by ticks (Nene et al. 1996, 1999; Musoke et al. 2005). A method of live vaccination known as infection and treatment (ITM) was developed approximately 40 years ago (Radley et al. 1975), and there is evidence that the protection induced by ITM is attributable primarily to class I major histocompatibility complex (MHC)-restricted CD8^+^ cytotoxic T cells (McKeever et al. 1994). However, this cytotoxic T cell response is strain-specific and strongly dependent on the bovine class I MHC phenotype of the host (Taracha et al. 1995). This may constrain development of a broadly cross-protective recombinant vaccine that mimics the cellular responses induced by ITM and highlights the potential importance of the conservation of the p67 antigen in cattle-derived *T. parva*. Extensive sequence divergence in the central region of the *p67* gene in buffalo-derived parasites from South Africa has recently been described (Sibeko et al. 2010). However, the p67 vaccine has not yet been tested in areas where the parasite challenge is mainly from *T. parva* originating from buffalo. We focus in the study described herein on in-depth analysis of p67 B cell epitope polymorphism in cattle-infective isolates from a specific geographical locality in central Kenya where buffalo and cattle co-graze. In addition, we examine whether any codons show signatures of positive selection in the central region of the *p67* gene.

## Materials and methods

### Parasite isolates and genomic DNA extraction

Genomic DNA preparations were made from 18 cryopreserved pellets of 10^7^ *T. parva* schizont-infected lymphocyte cultures initially isolated from cattle that co-grazed with the African Cape buffalo, using the DNeasy® Tissue Kit (Qiagen, Germany) according to the manufacturer’s instructions. The cattle were part of a field trial of ITM vaccines performed in the year 2000 to explore protection afforded to immunized animals that received a *T. parva* challenge from buffalo-associated ticks at Marula farm, central Kenya (Pelle et al. 2011). The trial was carried out in strict accordance with the recommendations in the standard operating procedures of the ILRI’s Institutional Animal Care and Use Committee (IACUC). Fifty-three of the 113 tick-exposed cattle developed clinical disease and died; mortality was observed in 40 immunized animals and 13 of the control cattle. Most animals exhibited clinical and parasitological features typical of those induced by buffalo-derived *T. parva* (Norval et al. 1992) with a low schizont parasitosis and low or, in some cases, no piroplasm parasitemia. As in Table 1, among the 18 cattle from which schizont-infected lymphocyte cultures were generated from lymph node biopsies were nonimmunized controls, as well as those immunized with one of the following stabilates: *T. parva* Marikebuni stabilate 3014 (Morzaria et al. 1995), *T. parva* Marikebuni stabilate 316 (Payne 1999), and *T. parva* composite trivalent Muguga cocktail stabilate FAO1 (Morzaria et al. 1999). All cattle were monitored daily from day 17 after exposure, and the clinical reactions are summarized in Table 1.

**Table 1:**
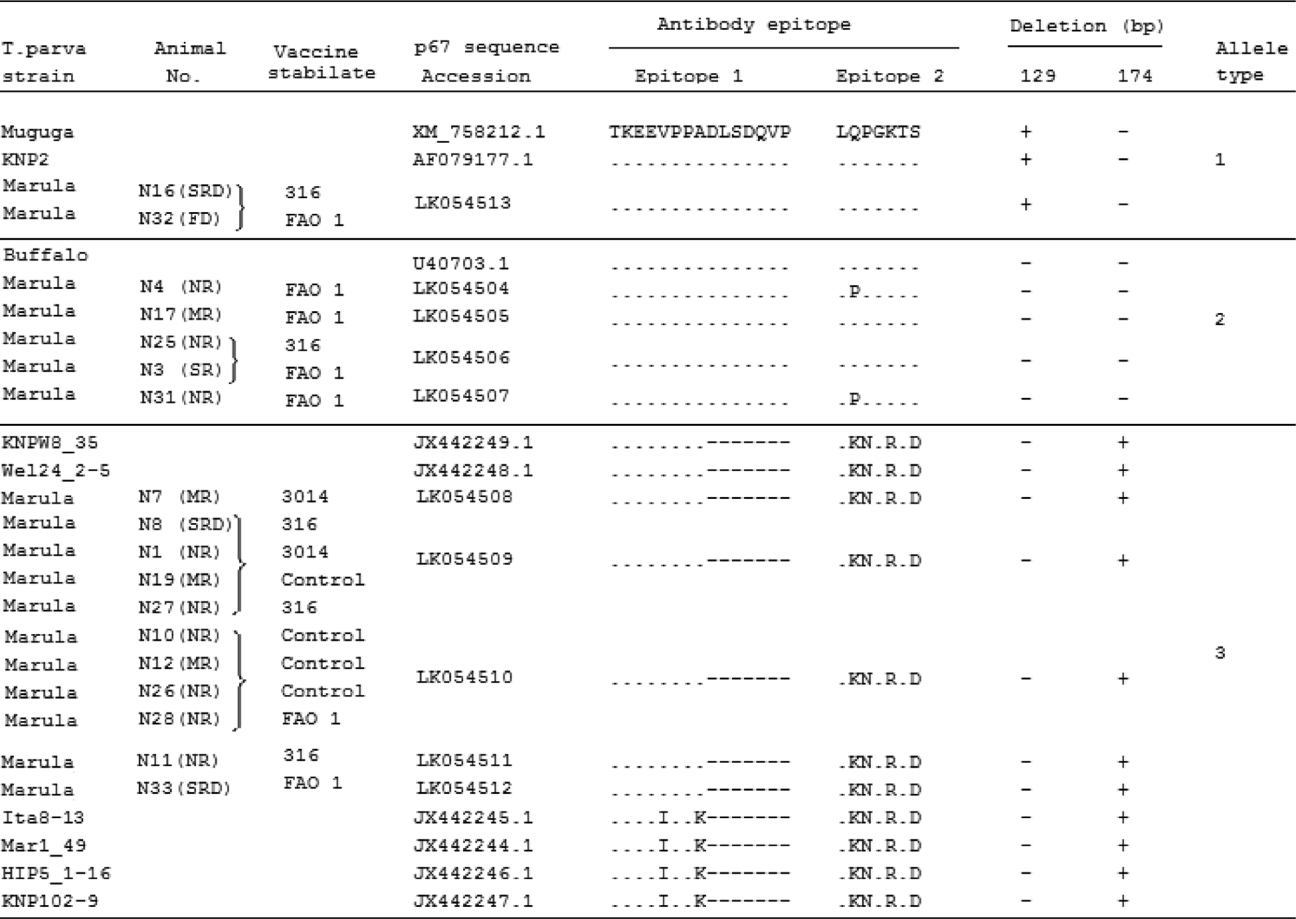
Classification of *T. parva* p67 alleles based on indels and B cell epitope sequence variation

Clinical reactions of cattle exposed to tick challenge at Marula farm are in parenthesis and abbreviated as follows: *SRD* severe reaction and died, *SR* severe reaction, *MR* mild reaction, *NR* nonreactor, *FD* found dead. *Curly braces* enclose animals from which the same sequence type was obtained. “.” Epitope positions which have a conserved residue; “-” gapped positions in the antibody epitopes

### Amplification and sequencing of the gene encoding *T. parva* p67

The amplification of the gene encoding p67 was performed using genomic DNA templates with primer pairs IL 613 3 (ACAAACACAATCCCAAGTTC) and IL 792 2 (CCTTTACTACGTTGGCG), designed to amplify a 900-base pair (bp) internal fragment containing part of exon 1 and part of exon 2 separated by the 29-bp intron sequence (Nene et al. 1996). These conserved primers amplify multiple *p67* alleles (Nene et al. 1996; Sibeko et al. 2010). The polymerase chain reaction (PCR) reagents and cycling parameters were as described previously (Nene et al. 1996). The PCR products were purified after fractionation through 1.2 % agarose gels, using the QIAquick gel extraction kit (Qiagen). The amplicons were sequenced bidirectionally using IL 613 3 forward and IL 792 2 reverse primers using Sanger dideoxy technology. Sequences were assembled and edited using the CLC Genomics Workbench version 6.0.

### p67 linear epitope sequence alignments, variant calling, and sequence logo creation

Basic Local Alignment Search Tool (BLAST) version 2.2.29+ (Altschul et al. 1990) search against a local database containing a repertoire of *T. parva* isolates in East and South Africa assigned the Marula query nucleotide sequences based on alignment scores to one of the four previously identified *T. parva p67* allele categories. The 13 sequences derived from *T. parva p67* antigen gene included in the local database were retrieved using the BLAST utility of the NCBI nucleotide database and had GenBank accessions: XM_758212.1, U40703.1, AF079177.1, JX442251.1, JX442249.1, JX442247.1, JX442246.1, JX442245.1, AF079176.1, JX442248.1, JX442244.1, JX442250.1, and AF079175.1. The sequences included in the local database were used as references in a multiple sequence alignment with *p67* sequences from isolates genotyped in this study covering the polymorphic central region of the gene. The alignment was generated with MAFFT version 7.122 using the L-INS-i option (Katoh et al. 2002), with minor subsequent manual adjustments. The program Seq2Logo version 1.2 (Thomsen and Nielsen 2012) was used to create a Kullback-Leibler sequence logo by calculating the information content as a function of the observed and background probability of residues at each position in the alignment region spanning the mapped targets of host B cell responses. In addition, estimates of *p67* gene polymorphism, *π*, calculated as the average number of nucleotide differences per site, were generated with DnaSP version 5 (Librado and Rozas 2009).

### Selection of best-fit nucleotide substitution models for *T. parva* p67 evolution

The phylogeny of the *T. parva* p67 antigen gene sequences was inferred from an optimal subset of the alignment generated as described above. This alignment subset was determined by the number of residues in gap-free columns by the program MaxAlign version 1.1 (Gouveia-Oliveira et al. 2007). We performed a preliminary reconstruction of the phylogenetic relationships between sequences using a neighbor-joining (NJ) tree (Saitou and Nei 1987) under the Jukes and Cantor (JC) model. The NJ-JC tree served as the basis for calculating the likelihood scores for 56 different nucleotide substitution models with varying sets of substitution rate parameters capable of accommodating rate heterogeneity between sites. The best fitting model for the *p67* data was selected based on the Akaike information criterion (AIC = −2lnL + 2*K*), a function of the maximized log-likelihood (lnL), and the number of estimated parameters (*K*) for a model (Wagenmakers and Farrell 2004), using the program Modeltest version 3.06 (Posada and Crandall 1998).

### Assessing model fits by using AIC

The phylogeny of the *T. parva* p67 antigen gene sequences was estimated with the maximum likelihood (ML) criterion and the parameters from the best-fit nucleotide substitution model, using PAUP version 4 (Swofford 2002). Estimations of *d*_S_ and *d*_N_ were performed using the codeml program from the PAML package version 4 (Yang 2007). These estimates were obtained under two different models of evolution: the nearly neutral (M1) and the positive selection (M2) models. M1 assumes two categories of sites, one evolving under purifying selection (0 < *d*_N_/*d*_S_ < 1) and the other neutral (*d*_N_/*d*_S_ = 1). Model M2 adds to these an additional category of positively selected sites (*1* < *d*_N_/*d*_S_). Both models were fitted by allowing different nucleotide frequencies for each codon position. The AIC was used to seek the fitted model where the information loss based on the expected relative Kullback-Leibler distance as defined by AIC differences (Δ_i_) was minimal.

## Results

### *p67* gene variants among *T. parva* isolates from Marula ranch (central Kenya)

**Fig. 1:**
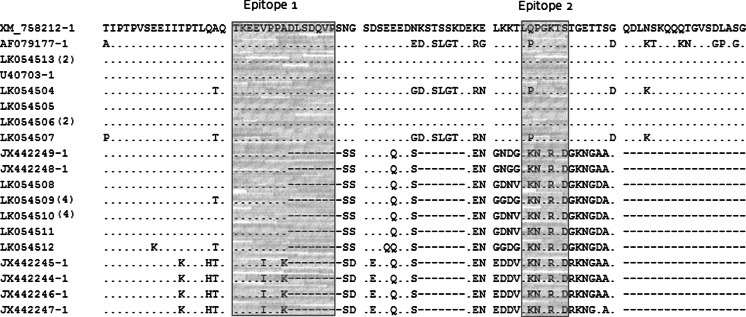
Multiple deduced amino acid sequence alignment of a section of the amplified internal fragment of the *T. parva p67* gene generated using the multiple sequence alignment program, MAFFT version 7.122 (Katoh et al. 2002) “.” Amino acid (aa) positions which have a conserved residue; “-” gapped aa positions; number of Marula isolates carrying an allele is indicated in *parentheses*. The epitopes recognized by anti-sporozoite monoclonal antibodies are *boxed*

We sequenced the central region of the *T. parva p67* gene from a collection of cultured schizont-infected lymphocytes recently isolated from cattle which had been exposed to tick challenge within Marula farm where the African buffalo co-grazed (Pelle et al. 2011). We generated an alignment of the amplified internal fragment of the *T. parva p67* gene, which contains 900 bp in the reference *T. parva* Muguga isolate. The alignment contains 27 sequences, nine of which are available in GenBank and 18 of which are derived from the Marula isolates (Fig. 1). There were ten different p67 sequences represented among the total of 18 from Marula, each of which was derived from a schizont culture isolate from a different animal (Table 1). None of the ten sequences was the same as the p67 sequences of the *T. parva* stocks within the immunizing stabilates, all of which are identical to one another (Nene et al. 1996, M. Norling and J. Silva in preparation). The Marula sequences described in this report are available from the European Nucleotide Archive at http://www.ebi.ac.uk/ena/data/view/LK054504-LK054513, and animals from which the same sequence type was obtained are shown in Table 1. Among the alleles sequenced from the Marula isolates, the overall DNA polymorphism, *π*, within and outside the mapped B cell epitopes in the amplified central fragment of the *p67* gene was 0.19627 (19.6 %). All sequences were divided into three allele types, 1 through 3, defined by the presence or absence of two indels (Table 1). The indel set comprised both a 129-bp deletion (allele type 1) and an additional 174-bp deletion (allele type 3). Fifty-seven percent of the sequences were within *p67* allele type 3 based on the indels and only 10 % were within allele size type 1. Approximately one third of all sequences in the Marula isolate collection were assigned to *p67* allele type 2 on the basis of sequence similarity to allele type 1 combined with the absence of the 129-bp deletion (Table 1).

### Sequence polymorphism between antibody epitopes within the *p67* gene for Marula isolates

We determined the degree of sequence variation within epitopes 1 and 2 of the *T. parva p67* gene in the Marula field isolates. The predicted protein sequences were compared with the previously characterized p67 protein sequences derived from East and South African isolates (Sibeko et al. 2010). Within the region encoding the mapped epitopes, the high level of nucleotide sequence polymorphism translated into coding changes that resulted in amino acid mutations relative to the *T. parva* Muguga reference allele (XM_758212.1), a cattle-type *p67* sequence (Nene et al. 1996). These variants are shown in Table 1. Among the sequences assigned to allele types 1 and 2, epitope 1 was remarkably conserved, perfectly matching the reference sequence. However, an in-frame deletion of a seven-amino acid segment was observed in a majority of the Marula sequences, all of which were assigned to allele type 3. This truncated epitope sequence resulting from the deletion appears to be a unique feature of the *p67* indel-defined allele type 3. In contrast, epitope 2 sequences were less homogeneous among the sequences assigned to allele types 1 and 2 due to a nucleotide substitution that conferred a predicted amino acid change, with the consensus glutamine, ^210^Q, being replaced by proline, P. Additionally, there were nonsynonymous mutations in epitope 2 relative to the reference sequence in all of the isolates assigned to *p67* allele type 3.

### Position-specific enrichment and depletion of residues within the p67 linear epitopes

**Fig. 2:**
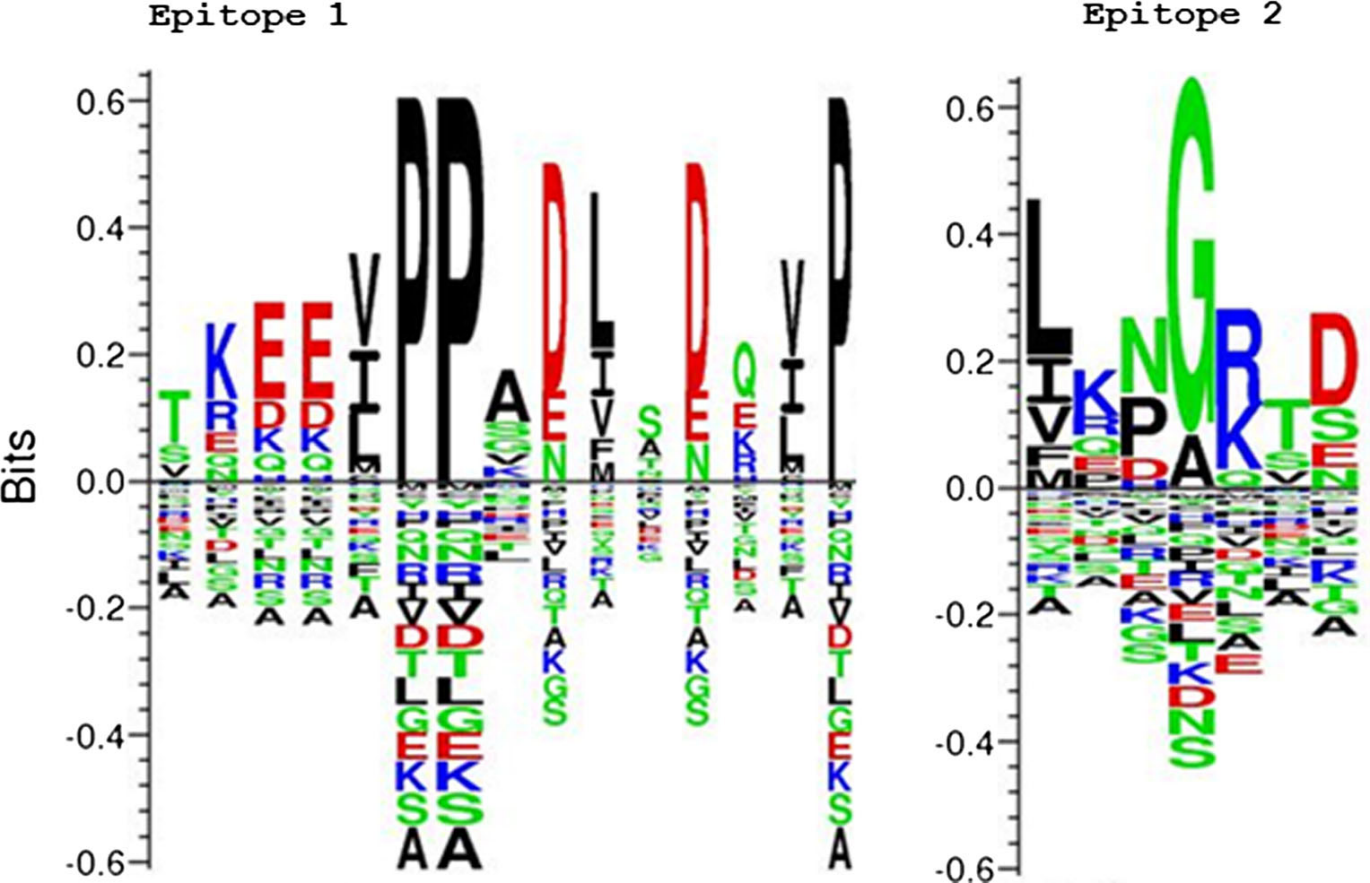
Kullback-Leibler logo for sequences corresponding to B cell epitopes 1 and 2 for sporozoite-neutralizing antibodies on the *T. parva* p67 based on a multiple alignment of the predicted amino acid sequences within the central region. Enriched amino acids (aa) are shown on the positive *y*-axis and depleted (underrepresented) amino acids on the negative *y*-axis. The height of the column of aa residues represents the level of conservation at a particular position, while the relative residue height denotes its frequency. Polar aa is depicted in *green*, basic aa in *blue*, acidic aa in *red*, and hydrophobic aa in *black*

Information content, a function of the observed and background probability for a particular column in the multiple sequence alignment, was calculated for each position spanning the mapped epitopes to assess their relative contributions to epitope polymorphisms and visualized in a Kullback-Leibler logo. As can be seen in Fig. 2, Seq2Logo (Thomsen and Nielsen 2012) captures the extent of B cell epitope polymorphism in *T. parva p67* among field isolates by taking advantage of pseudo count estimates and sequence weighting to deal with data redundancy and the low number of observations, respectively. The amino acids enriched at each epitope position are shown on the positive *y*-axis, and the corresponding depleted amino acids are on the negative *y*-axis.

### Analysis of Marula *p67* sequences revealed an excess of nucleotide substitutions resulting from transitions relative to transversions

To investigate the nature of diversity in the sequenced *p67* alleles, the model of nucleotide substitution that represents the best fit to the data, to be used for phylogenetic inference, was established by statistical testing. This was assessed by the AIC, a function of the maximized log-likelihood (lnL), and the number of estimated parameters (*K*) for a model. The *p67* dataset was best explained by the TIM+I+G model of evolution. This model assumes variable base frequencies, variable transition rates, two transversion rates, a proportion of invariable sites (I), and a gamma distribution with shape parameter (*α*). With the corresponding base frequencies taken into consideration, maximum likelihood estimates of substitution rates revealed more transitions than transversions. In particular, the A-to-G transition occurred most frequently; 65 % of the sites were invariable (*p-inv*), and the gamma shape parameter (α) was less than 1, the latter indicating a relatively slow rate of evolution at the *p67* locus (Table 2).

**Table 2:** AIC model selection and maximum likelihood estimates from best-fit model (TIM+I+G)

AIC nucleotide substitution model selection						
Model	lnL	*K*	AIC	Delta	Weight	Cum weight
TIM+I+G	3243.4773	8	6502.9546	0.0000	0.2086	0.2086
TrN+I+G	3244.5530	7	6503.1060	0.1514	0.1934	0.4021
K81uf+I+G	3245.0901	7	6504.1802	1.2256	0.1130	0.5151
HKY+I+G	3246.1763	6	6504.3525	1.3979	0.1037	0.6188
TIM+G	3245.1899	7	6504.3799	1.4253	0.1023	0.7211
TrN+G	3246.3713	6	6504.7427	1.7881	0.0853	0.8066
K81uf+G	3246.7419	6	6505.4839	2.5293	0.0589	0.8645
HKY+G	3247.9426	5	6505.8853	2.9307	0.0482	0.9136
TIM	3313.7852	6	6639.5703	136.6157	4.50e−31	1.0000
K80	3358.1204	1	6718.2407	215.2861	0.00e+00	1.0000
JC	3368.9949	0	6737.9897	235.0352	0.00e+00	1.0000
Maximum likelihood estimate from best-fit model (TIM+I+G)						
Base frequencies		Among-site rate variation				
freqA = 0.3548		Proportion of invariable sites (I) = 0.3332 Variable sites (G) Gamma distribution shape parameter = 06479				
freqC = 0.2131						
freqG = 0.2104						
freqT = 0.2216						
Substitution model						
Rate matrix						
R (a) [A-C] = 1.0000		R (b) [A-G] = 2.5858			R (c) [A = T] = 1.3319	
R (d) [C-G] = 1.3319		R (e) [C-T] = 1.7247			R (f) [G-T] = 1.0000	

The Akaike information criterion (AIC), a function of the maximized log-likelihood (lnL), and the number of estimated parameters (*K*) used to select the best fitting model. Delta denotes the AIC differences. Also shown are the maximum likelihood estimates of base frequencies, nucleotide substitution rates, and rate heterogeneity parameters (proportion of invariable sites and shape parameter of the gamma distribution)

### Spatial clustering of positively selected codons in the *p67* gene

We made maximum likelihood inferences of positive selection based on the AIC approach to compare the fit of models M1, the quasi-neutral model, which allows for two site classes (*d*_N_/*d*_S_ = 1, 0 < *d*_N_/*d*_S_ < 1) relative to a positive selection model, M2, with three site classes (*d*_N_/*d*_S_ = 1, 0 < *d*_N_/*d*_S_ < 1, and *d*_N_/*d*_S_ > 1). As shown in Table 3, fitting the codon substitution model that captures the proportion of codons for which *d*_N_/*d*_S_ > 1 within the *p67* gene provided a better fit than M1 (minimum AIC value). In the best fit model (M2), about 53 % of the codons are strongly conserved (average *d*_N_/*d*_S_ = 0.11037), 35 % are consistent with neutral evolution (*d*_N_/*d*_S_ = 1.0), and the other 10 % of codons exhibit *d*_N_/*d*_S_ values suggesting evolution under positive selection (average *d*_N_/*d*_S_ = 2.95491). Bayesian posterior probabilities calculated using Bayes empirical Bayes (BEB) (Yang et al. 2005) in M2 revealed the codons with signatures of positive selection (Table 4). However, in the case of most codons, the standard error interval includes *d*_N_/*d*_S_ = 1, and the posterior probability of any codon evolving with *d*_N_/*d*_S_ > 1 is never equal to, or larger than, 95 % and therefore not significant at this level.

**Table 3:** Parameters and Akaike information criterion (AIC) scores of the *p67* gene under codon site models

Model	*K*	lnL	AIC_c_	Δ_*i*_	*d*_N_/*d*_S_ < 1	*d*_N_/*d*_S_ = 1	1 < *d*_N_/*d*_S_
M1	49	1260	2618	2.0	*p* 0.54325	*p* 0.45675	
					*ω* 0.09055	*ω* 1.00000	
M2	51	1257	2616	0.0	*p* 0.53538	*p* 35855	*p* 0.10607
					*ω* 0.11037	*ω* 1.00000	*ω* 2.95491

The Akaike information criterion (AIC), a function of the maximized log-likelihood (lnL), and the number of estimated parameters (*K*) used to select the best fitting model. *ω* is the Akaike weight and *p* gives the proportion of codon sites belonging to each of the *d*_N_/*d*_S_ ratio classes, and Δ_i_ denotes AIC differences

**Table 4:** Posterior probabilities (Pr) and estimated *d*_N_/*d*_S_ ratios (post mean ± SE for *d*_N_/*d*_S_) calculated using Bayes empirical Bayes (BEB) analysis for sites likely to be under positive selection on the *T. parva p67* gene identified using the reference stock *T. parva* Muguga

Positively selected site	Amino acid	Pr (*d*_N_/*d*_S_ > 1)	Post mean ± SE for *d*_N_/*d*_S_
151	T	0.645	2.257 ± 1.363
186	G	0.868	2.815 ± 1.394
194	N	0.686	2.259 ± 1.212
204	E	0.532	1.893 ± 1.094
205	L	0.545	1.907 ± 1.080
208	T	0.772	2.505 ± 1.294
237	L	0.801	2.596 ± 1.323
262	G	0.535	1.871 ± 1.047
277	H	0.745	2.469 ± 1.335
278	Q	0.671	2.247 ± 1.243
280	V	0.654	2.238 ± 1.291

## Discussion

We present evidence for *p67* alleles grouped into three distinct indel types within a single *T. parva* population infecting cattle that were part of a field trial of ITM vaccines designed to evaluate protection afforded to immunized animals that received a *T. parva* challenge from buffalo-associated ticks (Pelle et al. 2011). All of these p67 alleles were derived from *T. parva* genotypes, present in the Marula tick population, since although ITM immunization typically induces a persistent carrier state in cattle, both Marikebuni and the Muguga, Serengeti and Kiambu V stocks within the trivalent FAO1 Muguga cocktail, have an identical p67 sequence that differs from the p67 sequences of any of the Marula isolates. Comparable levels of heterogeneity at the *T. parva p67* locus have recently been reported in South Africa (Sibeko et al. 2010). Classification relative to previously described alleles was based on presence or absence of 129- and 174-bp indels in the central region of the *p67* gene. We show that parasites with a 174-bp deletion (allele type 3) were most frequent in the isolates from the cattle under challenge at Marula farm. Whether there is any underlying selective advantage that the deletion might confer is unclear. One possibility is that epitope 1 is truncated and therefore not recognized in parasites containing the 129-bp sequence but with the 174 bp deleted.

The ten different *T. parva p67* sequences described herein (derived from a total of 18 sequences) were distinct at a minimum of 144 nucleotides excluding sequence reads covering indels. This contrasts strongly with the complete conservation of nucleotides at the *p67* locus, including introns and the third base in codons, in cattle-transmissible parasites (Nene et al. 1996, Musoke et al. 2005). We assessed the nature and extent of variation in the two closely juxtaposed B cell epitopes that have been mapped to the central polymorphic region of the p67 protein (Nene et al. 1999). Our analysis demonstrated that all allele type 1 sequences containing the 174-bp sequence but lacking the 129-bp sequence were identical to the prototype Muguga (XM_758212.1) at both epitopes analyzed. This was also the case for the isolates within the indel-defined allele type 2 with the exception of a single variation present in two isolates. The high level of conservation of these epitopes suggests that they are candidates for inclusion in multivalent vaccines with the potential to reduce establishment of infection when challenge occurs with multiple *T. parva* genotypes. Indeed, neutralizing antibodies directed against conserved epitopes have been found to be correlates of protection against infective sporozoites in several species of parasitic protozoans within the phylum apicomplexa including *Plasmodium* (Weedall et al. 2007), *Eimeria* (Wallach et al. 2008), and *T. parva* (Musoke et al. 1992).

Although studies with monoclonal antibodies have defined ^169^ TKEEVPPADLSDQVP ^183^ as a p67 epitope in *T. parva* isolates that are transmissible between cattle by ticks (Nene et al. 1999), our analysis reveals a deletion variant in this region that is common to all type 3 alleles, suggesting that it may not represent a B cell epitope in many buffalo-derived *T. parva* parasites. Interestingly, there is a high level of conservation of the epitope sequence flanking the deletion in the isolates carrying allele type 3. The amino acid sequence of epitope 2 in allele 3 isolates is also conserved but distinct from that of allele types 1 and 2, although not altered in length. Collectively, these findings support the hypothesis that these *p67* regions are potential targets for inclusion in a combinatorial vaccine formulation that includes immunodominant B cell epitopes. Vaccine strategies similar to those being pursued for the polymorphic merozoite surface protein (MSP) 1 malaria protein could also be applicable to *T. parva*. In the case of MSP 1, it has been suggested that focusing on both the conserved C-terminal region (Blackman et al. 1994) in combination with polymorphic subtype-restricted epitopes (Tetteh et al. 2005) to create chimeric constructs could be a viable vaccine development strategy. For *T. parva* p67, a vaccine could also incorporate conserved epitopes located outside the p67 central region, for example, in the C-terminal region in which no polymorphism has been reported, within a single chimeric construct.

We used the multiple sequence alignment generated from the *p67* dataset described in Table 1, to investigate what modality of selection may have contributed to the evolution of the *p67* gene. Such tests typically assume a particular phylogeny and can be influenced by the nucleotide substitution model selected (Yang et al. 1994). After optimization of the parameters, an AIC analysis indicated that the TIM+I+G model of substitution represented the best fit to the *p67* polymorphism data. AIC is an efficient alternative to likelihood ratio tests (Posada and Buckley 2004) and has recently been implemented in the identification of the codon substitution model that best fits sequence polymorphisms in a region of VAR2CSA—a *Plasmodium falciparum* protein involved in placental sequestration in the mammalian host (Dahlbäck et al. 2006). We detected a subset of codons with signatures of positive selection using codon-based likelihood analysis based on the observed *d*_N_/*d*_S_ rate ratio (Yang and Bielawski 2000). The parameters defining the codon evolution model used in this study were derived by capturing heterogeneity of *d*_N_/*d*_S_ across multiple sites, with positive selection inferred when *d*_N_/*d*_S_ > 1. Under the codon model that provided the best fit to the data, model M2, about 10 % of codons showed potential evidence of positive selection (average *d*_N_/*d*_S_ = 2.95). However, despite the demonstration of evolutionary patterns consistent with the effects of positive selection, none of the amino acid sites in gap-free alignment columns in the *p67* gene identified by maximum likelihood analysis had a highly significant (>95 %) posterior probability of being positively selected (Table 4). It is possible that the high *d*_N_/*d*_S_ ratio observed for several amino acids results from allelic dynamics and does not accurately reflect the effect of selection (Kryazhimskiy and Plotkin 2008). Interestingly, none of the codons located within defined linear epitopes recognized by monoclonal antibodies shows signatures of positive selection. These results are consistent with a recent study that showed no preferential distribution of sites under positive selective pressure within T cell epitopes in candidate vaccine antigens from the pathogenic *T. parva* schizonts (Pelle et al. 2011). Among the 115 cattle exposed to challenge at Marula, from which the 18 isolates analyzed were derived, approximately 80 % of those that developed severe disease and died exhibited features associated with corridor disease, specifically low schizont parasitosis and piroplasm parasitemia, irrespective of whether they were isolated from vaccinated or control cattle (R. Bishop and A. Musoke, unpublished data). One conclusion from this study is therefore that *T. parva* parasites originating from buffalo can infect and cause severe disease in co-grazing cattle and have diverse p67 genotypes, unlike the conserved p67 sequence observed in *T. parva* populations that have adapted to transmission between cattle. The data also suggests that buffalo-derived *T. parva* can sometimes “break through” the immunity induced by ITM immunization and induce severe clinical reactions, in areas with a high tick challenge.
